# Antiviral Compounds Screening Targeting HBx Protein of the Hepatitis B Virus

**DOI:** 10.3390/ijms231912015

**Published:** 2022-10-10

**Authors:** Yaojia Ma, Shingo Nakamoto, Junjie Ao, Na Qiang, Tadayoshi Kogure, Keita Ogawa, Miyuki Nakagawa, Kisako Fujiwara, Terunao Iwanaga, Ryuta Kojima, Hiroaki Kanzaki, Keisuke Koroki, Kazufumi Kobayashi, Naoya Kanogawa, Soichiro Kiyono, Masato Nakamura, Takayuki Kondo, Ryo Nakagawa, Sadahisa Ogasawara, Ryosuke Muroyama, Tetsuhiro Chiba, Jun Kato, Naoya Kato

**Affiliations:** Department of Gastroenterology, Graduate School of Medicine, Chiba University, Chiba 260-8670, Japan

**Keywords:** Hepatitis B virus, HBx protein, SPRi screening, antiviral, HBx inhibitor

## Abstract

A functional cure of hepatitis B virus (HBV) infection or HB antigen loss is rarely achieved by nucleos(t)ide analogs which target viral polymerase. HBx protein is a regulatory protein associated with HBV replication. We thought to identify antiviral compounds targeting HBx protein by analyzing HBx binding activity. Recombinant GST-tagged HBx protein was applied on an FDA-approved drug library chip including 1018 compounds to determine binding affinity by surface plasmon resonance imaging (SPRi) using a PlexArray HT system. GST protein alone was used for control experiments. Candidate compounds were tested for anti-HBV activity as well as cell viability using HepG2.2.15.7 cells and HBV-infected human hepatocytes. Of the 1018 compounds screened, 24 compounds showed binding to HBx protein. Of the top 6 compounds with high affinity to HBx protein, tranilast was found to inhibit HBV replication without affecting cell viability using HepG2.2.15.7 cells. Tranilast also inhibited HBV infection using cultured human hepatocytes. Tranilast reduced HB antigen level dose-dependently. Overall, theSPRi screening assay identified novel drug candidates targeting HBx protein. Tranilast and its related compounds warrant further investigation for the treatment of HBV infection.

## 1. Introduction

Hepatitis B virus (HBV) infection causes chronic infection and risk of liver cirrhosis and hepatocellular carcinoma [1]. Current anti-HBV drugs, such as nucleos(t)ide analogs, are effective in suppressing viral replication by inhibiting HBV polymerase function and can slow the progression of the disease [2]. However, off-therapy usually results in viral rebound and relapse of the hepatitis, and thus long-term treatment is required. The current treatment goal for HBV infection is to achieve a functional cure of the condition, specifically sero-clearance of the hepatitis B surface antigen [2]. Nucleos(t)ide analogs rarely achieve this goal, however; thus, new drugs based on a different mechanism of action have long been anticipated. Currently, there has emerged a new class of anti-HBV inhibitors such as HBV capsid inhibitor and silencing RNAs, which are under evaluation in clinical trials [3]. In addition to these, targeting covalently closed circular DNA (cccDNA) is an attractive approach albeit still in the preclinical stage.

HBV are classified into 9 to 10 major genotypes (A-J) [4]. Of these, genotype A-D has been the most studied; genotype B and C are common in East Asia, while genotype A and D tend to be prevalent in Western countries. HBV genome encodes 7 proteins, secretory protein (preCore), nucleocapsid (core), polymerase (pol), HBx and 3 envelope proteins, L, M, and S7. Among them, HBx is a regulatory protein that is required for efficient transcription of cccDNA, but its precise role is not yet understood [5]. HBx is a 154 amino acid protein that can activate a variety of cellular and viral promoters and act on many DNA binding motives including binding sites for NF-kB and AP-1. Indeed, HBx is reported to interact with several transcription factors and activate signal pathways, which has been associated with efficiency in HBV replication [5].

It has been shown that an HBx-defective virus cannot replicate well in an HBV infection cell culture model and an HBV infection mouse model [6,7]. A recent report suggested that the role of HBx on HBV replication relies on interaction with DDB1, a component of the Cullin 4 (CUL4) ubiquitin E3 ligase complex [8]. Specifically, amino acid 88–100 of HBx is shown to bind to DDB1 [9]. Other reports indicate that B-cell lymphoma 2 (Bcl-2) Homology 3 (BH3)-like motif in amino acid 113–135 of HBx, is associated with HBV replication [10]. HBx have also been shown to target cccDNA to affect the formation of cccDNA as well as viral transcription [8,11,12].

Because HBV is a hepatotropic virus with strict specificity for human hepatocytes, there are limited cell systems for studying it [13]. Human hepatoma cell lines have been widely used for studying HBV replication, although they do not support HBV infection. Hepatoma cell lines expressing sodium taurocholate co-transporting polypeptide, known as HBV receptor, can support HBV infection. These cells are transformed and only partially mimic normal hepatocytes. Primary human hepatocytes can most closely mimic normal hepatocytes. However, infection efficiency varies among different donors.

Because HBx has multiple functions and has been difficult to purify [14], reports on drug discovery targeting HBx protein have been limited. Pathak et al. [15] used in silico predicted HBx structure for docking analysis against natural compounds and found rutin derivatives as a candidate compound for binding with HBx. Sekiba et al. [16] screened compounds targeting HBx-DDB1interaction by the split luciferase assay system and found nitazoxanide as a candidate anti-HBV compound. These reports indicate that HBx have potential to be an antiviral drug target.

In this study, we thought to identify HBx-binding compounds which inhibit viral replication by disrupting HBx’s function. A previous study has successfully identified a small molecule that bound to a specific protein and disrupted the function of the protein by a chemical array method [17]. We used an array of FDA-approved compounds for screening and measured HBx-binding ability using surface plasmon resonance imaging (SPRi) assay [18]. Candidate compounds that bound themselves to HBx were assessed for antiviral activity using cell culture infection models.

## 2. Results

### 2.1. GST-HBx Expression

**Figure 1:**
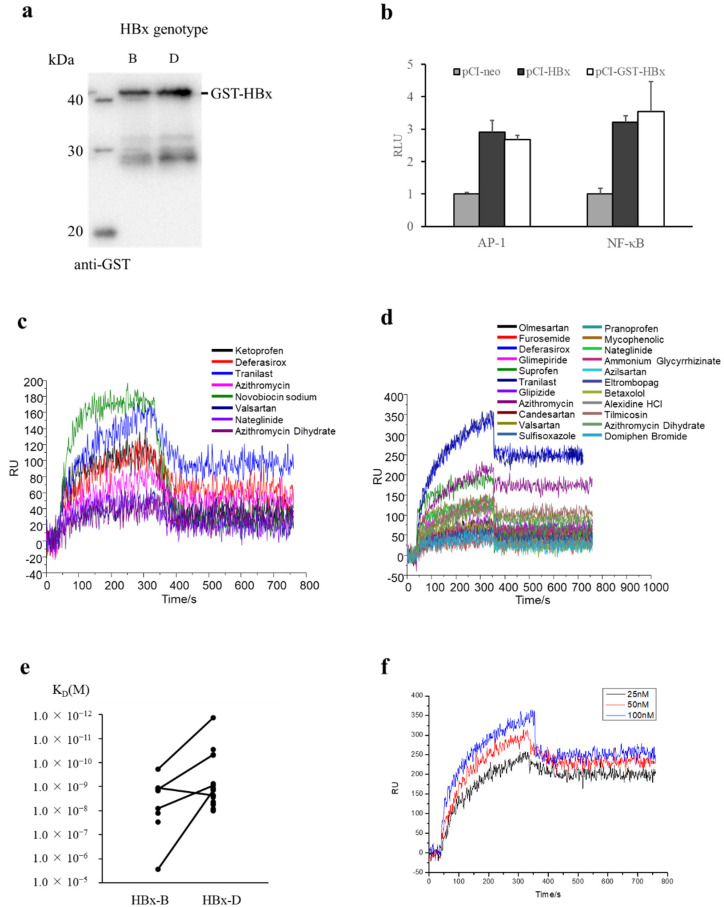
Screening of GST-HBx binding compounds. (**a**), Immunoblot of purified GST-HBx (genotype B) and GST-HBx (genotype D). (**b**), Transactivation function of GST-HBx. HeLa cells were transfected with HBx or GST-HBx as well as indicated reporter vector and Rluc control vector for correction. After 24 h, luciferase assay was performed. The assay was performed in triplicate and expressed as mean and standard deviation. The value of control cells without HBx (pCI-neo) was set as 1. Representative results are shown from one of three independent experiments. (**c**), SPRi sensogram showing positive binding hits of GST-HBx (genotype B) protein (8 hits). (**d**), Positive binding hits of GST-HBx (genotype D) protein (22 hits). (**e**), KD values of hit compounds between genotype B HBx and genotype D HBx are compared. The same compounds are connected with lines. (**f**), Serial dilutions of GST-HBx (genotype D) protein binding to top hit compound, tranilast. HBx-B, genotype B HBx; HBx-D, genotype D HBx.

Because purification of HBx alone was difficult due to insolubility [14], HBx was expressed as a GST-fused protein. The expression and purification of GST-HBx-B, derived from HBV genotype B, or GST-HBx-D, derived from the HBV genotype D, were confirmed by CBB staining and immunoblot showing the band with the expected size (GST 26 kDa; GST-HBx 43 kDa; Figure 1a, Supplementary Figure S1a).

### 2.2. Transcriptional Activity of GST-HBx

Because GST protein is larger than HBx protein (26 kDa vs. 17 kDa), we examined if GST tag affected HBx protein function. HBx has been shown to have a transcriptional activating function to promote HBV repliation [19,20]. Transcriptional activity of GST-HBx was compared to that of HBx alone by AP-1 or NF-kB luciferase assay (Figure 1b). HBx and GST-HBx induced AP-1 or NF-kB promoter activity to the same degree, indicating that GST-tag had little effect on the transactivation activity of HBx.

### 2.3. SPRi Screening to Target HBx Protein

The binding of GST-HBx protein to 1018 FDA-approved compounds was screened on a chip by SPRi assay. Using genotype B HBx protein, 8 compounds were identified as HBx binding compounds (Figure 1c, Supplementary Table S1), while genotype D HBx protein bound to 22 compounds (Figure 1d, Supplementary Table S2). Of these, 5 compounds bound to both proteins from genotype B HBx and genotype D HBx (Figure 1e), resulting that 25 compounds were identified as candidate compounds showing HBx binding ability. There were 6 compounds with a dissociation constant of less than 1 nM, showing their higher binding affinity (Table 1, Figure 1f, Supplementary Figure S1b–h). Of these, tranilast showed the highest binding affinity to both proteins from genotype B HBx and genotype D HBx (Figure 1f, Supplementary Figure S1b). The other 5 compounds showed binding affinity to genotype D HBx. These six compounds were further investigated.

### 2.4. Anti-HBV Activity of Candidate Compounds

**Figure 2:**
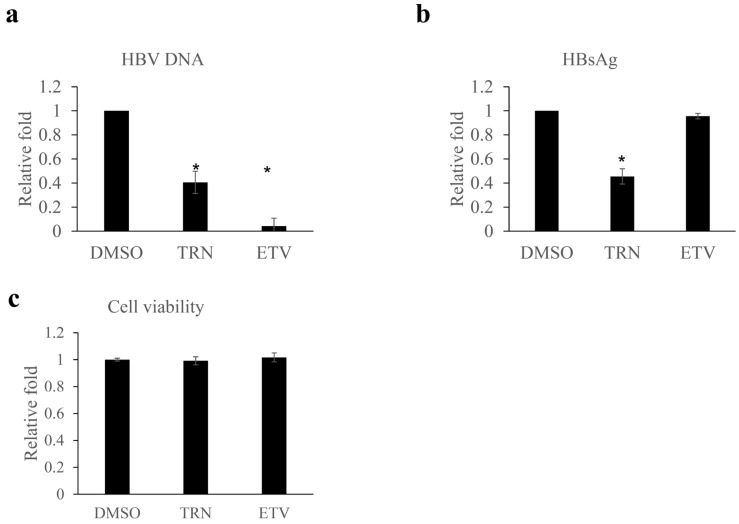
Effect of tranilast on anti-HBV activity in HepG2.2.15.7 cells. (**a**) Cells were treated with each compound at the concentration of 10 μM for 9 days. HBV DNA in the medium was quantified by real-time PCR. The value of DMSO control was set as 1. (**b**) HBsAg in the medium was quantified by CLEIA. (**c**) Cell viability was determined by cell proliferation assay. Experiments were performed in triplicate and expressed as means and standard deviation. * *p* < 0.05 compared to control. ETV, entecavir; TRN tranilast.

The anti-HBV activity of HBx-binding compounds was assessed using an HBV (genotype D) replicating liver tumor cell line, HepG2.2.15.7 cell (Supplementary Figure S2). Tranilast showed the highest binding affinity and showed antiviral activity. Tranilast is an anti-allergic drug and plasma concentration after the therapeutic dose of the drug reaches 30–300 μM [21]. Nucleoside analog, entecavir treatment decreased HBV DNA to less than 10% of the treatment control, while HBsAg level was not affected. In contrast, 10 μM of tranilast both decreased the HBV DNA and HBsAg level without affecting cell viability (Figure 2).

### 2.5. Anti-HBV Activity of Tranilast on Human Hepatocytes

**Figure 3:**
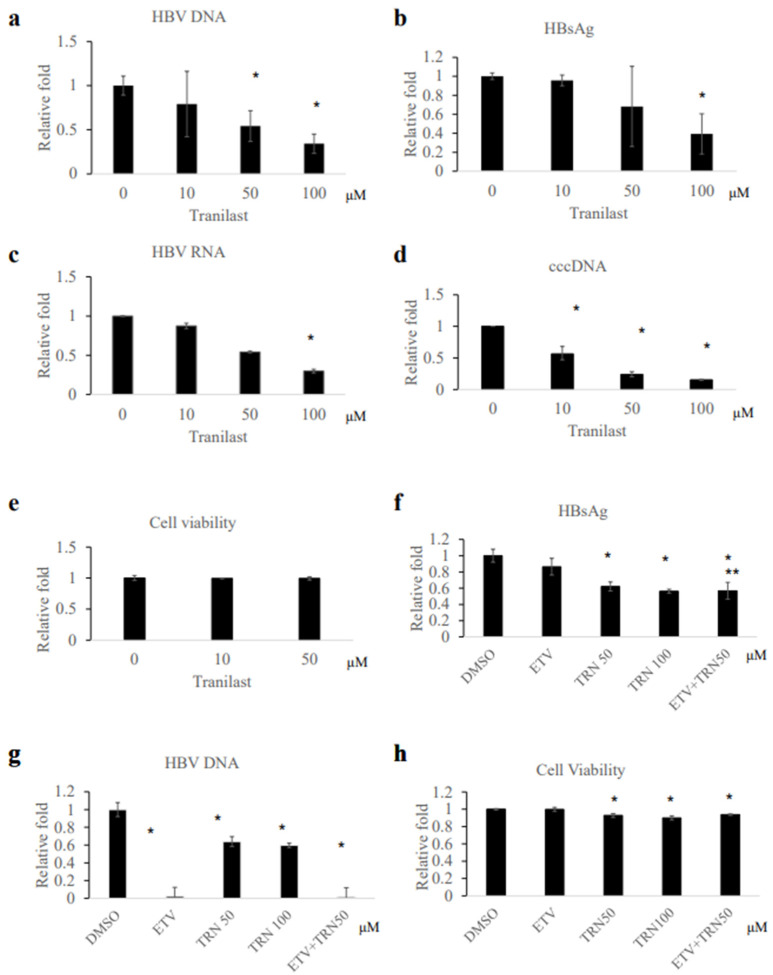
Effect of tranilast on HBV infection in human hepatocytes. (**a**–**d**) Human hepatocytes, PXB cells were infected with HBV and treated with the compound at the indicated concentration. At day 7, HBV DNA in the medium, HBsAg in the medium, intracellular HBV RNA level, and intracellular cccDNA level were measured. Experiments were performed in duplicate. (**e**) Cell viability on day 7 was determined by cell proliferation assay. Data were expressed as mean and standard deviation (*n* = 4). (**f**,**g**) and PXB cells were infected with HBV and cultured. On day 14, the indicated concentration of tranilast or entecavir (0.1 μM) was added for 7 days. Note that the peak plasma concentration of entecavir is 0.02–0.04 μM in the clinical setting [22]. On day 21, the HBsAg level and HBV DNA level in the culture medium were determined. The data were expressed as mean and standard deviation (*n* = 4). (**h**) Cell viability on day 21 was determined by cell proliferation assay. The data were expressed as mean and standard deviation (*n* = 4). * *p* < 0.05 compared to the control. ** *p* < 0.05 compared to entecavir alone. ETV, entecavir; TRN, tranilast.

Tranilast was further investigated for antiviral activity using HBV-infected human hepatocytes (Figure 3). Tranilast treatment was initiated at the time of HBV (genotype C) infection and continued for 7 days. Tranilast treatment decreased HBV DNA and HBsAg in the culture medium in a dose-dependent manner (Figure 3a,b). However, it should be noted that the changes in HBV DNA and HBsAg level did not exceed 10-fold. HBV RNA and cccDNA in the infected cells were also decreased by the compounds (Figure 3c,d). No obvious cell cytotoxicity was observed (Figure 3e). These results indicate that tranilast could inhibit HBV infection on human hepatocytes. When tranilast treatment started later after infection (>14 days), at which time entecavir could not effectively inhibit HBsAg production in contrast to potent suppression of HBV DNA, anti-viral effect was still observed (Figure 3f,g). Furthermore, when entecavir treatment was added along with tranilast, HBsAg reduction was greater than entecavir treatment alone (Figure 3f, Supplementary Figure S3). Cell viability assay showed no remarkable cytotoxicity under the condition (>90% activity compared to control).

### 2.6. The Effect of Tranilast on HBx-Induced Transcriptional Activity

The transcriptional activity of HBx has been important for HBV replication [20]. Because tranilast showed binding interaction with HBx, the effect of the compound on HBx’s function was studied. HepG2 cells were transfected with HBx expression vector and AP-1 or NF-kB luciferase reporter and then treated with or without tranilast. Tranilast treatment tended to inhibit HBx-induced AP-1 or NF-kB promoter activity, although the effect was not significant. Overall, tranilast had little impact on HBx-induced AP-1 or NF-kB promoter activity (Supplementary Figure S4).

## 3. Discussion

Using an FDA-approved drug library, the present study identified 24 compounds with ability to bind HBx protein (Figure 1, Supplementary Figure S1). Because HBx has been known to play an important role in HBV replication [19,20], compounds that bind to HBx protein would have antiviral activity. Among the HBx-binding compounds, tranilast showed ability to inhibit HBV replication. The concentration of tranilast was positively associated with the binding activity of HBx and with HBV inhibition activity (Figure 1f and Figure 3).

Tranilast treatment inhibited HBV infection with a decrease in HBV DNA as well as HBsAg and cccDNA in HBV-infected human hepatocytes (Figure 3a–d). Tranilast was effective in decreasing HBs antigen level when added at a later time point after HBV infection, but entecavir was not (Figure 3f), suggesting that tranilast inhibited HBV infection via a different mechanism from entecavir. We examined if tranilast affected the transactivation function of HBx. However, AP-1 and NF-kB reporter activity was not significantly affected (Supplementary Figure S4). Because HBx is a multifunctional protein, other mechanisms could be involved in the inhibition of HBV replication by tranilast. It is theoretically possible that inhibiting HBx’s function reduced viral antigen production, because HBx have roles in regulating transcription from cccDNA and in the formation of cccDNA [8,11,12]. As of now, it remains to be confirmed whether the anti-HBV effect of tranilast is through binding to HBx protein, and this question needs further research.

Tranilast has been used as an anti-allergic drug which is a synthetic analog of tryptophan metabolite [21]. It has been used in the treatment of inflammatory disease and several disease conditions including fibrosis and cardiovascular diseases and cancers. However, few studies reported an antiviral role for tranilast [23]. Although the concentration of tranilast used for the anti-HBV assay in this study is within the peak concentration in plasma after therapeutic dosage in patients (30–300 μM) [24], a relatively high dose (50–100 μM) was required to exert anti-HBV activity using hepatocyte culture compared to the tumor cell line (Figure 2 and Figure 3, Supplementary Figure S3). It is possible that the different mechanism of replication affected the results because HBV replication is mainly from integrated HBV DNA in the tumor cell line, while HBV replication is from cccDNA in human hepatocyte culture [25]. Another possibility is that the difference in HBV genotype (genotype D in HepG2.2.15.7 vs. Genotype C in hepatocyte) affected the results, although we do not have the data about the binding ability of the compound to HBx genotype C protein. Modification of the discovered compound will be needed to develop an antiviral drug with more potent activity. Among several compounds structurally related to tranilast, BIBR1532, non-competitive telomerase inhibitor, showed anti-HBV activity using human hepatocytes (Supplementary Figure S5).

Because we screened an FDA-approved library, candidate drugs have been used in clinical practice (Table 1). Repositioning existing drugs for a new indication has been an effective approach because it could reduce risk, development costs, and timelines [26]. On the other hand, the screening method can be applied to different arrays of compounds on a large scale, which would lead to the identification of more potent candidates. We are currently conducting another screening comprising more than 20,000 compounds. Of note, the current approach to screening could be applicable to identify anti-tumor drugs, because HBx has been associated with hepatocellular carcinoma development [5].

There are several limitations in this study. First, the findings have not been confirmed in vivo. Second, GST-tagged HBx protein was used instead of native HBx protein. Although GST-tag had little effect on transactivation activity of HBx (Figure 1b), using smaller protein tags or native HBx protein would be necessary for future screening experiments. Third, the specific binding region of HBx that interacts with drugs is not determined. Because purification of native HBx protein alone is difficult, only a small portion of HBx peptide have been cocrystallized with cellular protein such as DDB1 [9] and Bcl-2 family protein [10] so far. Future studies, such as a crystal structure experiment and inhibition of the interaction assay, are needed to confirm that tranilast actually binds HBx protein. It would be interesting to examine the role of tranilast in the HBx-DDB1 interaction [16]. Finally, the impact of HBx sequence variation on the binding ability of compounds to HBx protein has not been examined. There were 3 amino acid differences (1.9%) between genotype D HBx protein used for SPRi screening and that in HepG2.2.15.7 cells used for antiviral screening. In addition, human hepatocyte was infected with genotype C HBV. There were 12 amino acid differences (7.8%) between each HBx protein. Importantly, tranilast showed antiviral activity using HBx of both HBV genotypes.

In conclusion, SPRi screening using an array of FDA-approved drugs identified 24 compounds that bound to HBx protein. Of these, tranilast showed potent HBx binding activity as well as anti-HBV activity. Further studies will be needed to confirm if the drug can be used for the treatment of HBV infection with a new mode of action. The drug will also be used as a lead compound for future drug design of antiviral compounds. We showed that HBx protein could be targeted to discover new anti-HBV drugs.

## 4. Materials and Methods

### 4.1. Cell Culture

HeLa cells and HepG2 cells were obtained from the RIKEN cell bank (Ibaraki, Japan). The cells were cultured with a DMEM medium (Sigma-Aldrich, Tokyo, Japan) with 10% fetal bovine serum (Life technologies, Tokyo, Japan), 100 U/mL of penicillin, and 100 μg/mL of streptomycin (Life technologies). HepG2.2.15.7 cells were obtained from Dr. Wakita T (National Institute of Infectious Disease, Tokyo, Japan). The cells were cultured according to the method in the previous study [18]. Human hepatocytes produced from a chimeric mouse with humanized liver, and PXB cells were purchased from Phoenixbio (Hiroshima, Japan) and cultured according to the method in the previous study [25].

### 4.2. Compounds

The FDA1018 drug library was prepared by Plexera LLC (Tokyo, Japan). In addition, following chemicals were also used: entecavir (Santacruz, TX, USA), tranilast, n-(p-amylcinnamoyl) anthranilic acid, BIBR 1532 (Cayman chemical, MI, USA), cis-tranilast (Toronto research chemicals, Toronto, Canada), EN300-08353 (Enamine, Kyiv, Ukraine), and FT011 (Sigma-Aldrich).

### 4.3. Protein Expression, Purification, and Immunoblot

The construction of N-terminally GST-tagged HBx expression vector, pGEX-HBX was described previously and obtained from Dr. Tanaka Y (Tokyo University, Tokyo, Japan). The expression of GST-HBx protein was also described [27]. We prepared GST-HBx protein from genotype B, genotype C, and genotype D. Because GST-HBx protein from genotype C could not be expressed effectively, GST-HBx protein from genotype B (GenBank ID: AB246340) and genotype D (GenBank ID: AB104894) was used in subsequent experiments. Briefly, Escherichia coli BL21 (DE3) (Stratagene, La Jolla, CA, USA) was transformed by pGEX or pGEX-HBx, then induced with 1 mM isopropyl-β-D-thiogalactopyranoside for 3 h at 37 °C, pelleted, and lysed in phosphate-buffered saline containing 5 mM ethylenediaminetetraacetic acid, 1 mM dithiothreitol, 1 mM phenylmethylsulfonyl fluoride, 100 nM pepstatin, and 1% Triton X-100. After the sonication and centrifugation (8000× *g* for 10 min at 4 °C), the proteins were purified using glutathione–Sepharose 4B (Amersham Biosciences, Tokyo, Japan). The protein was separated by 10% sodium dodecyl sulfate-polyacrylamide gel electrophoresis and stained with Coomassie brilliant blue (CBB) or transferred to the Hybond-P polyvinylidene fluoride (Amersham Biosciences). The membrane was probed with anti-GST antibody (Wako-Junyaku, Osaka, Japan) and with anti-mouse horseradish peroxidase–conjugated secondary antibody, and then the signal was detected with ECL-plus (Amersham Biosciences).

### 4.4. Luciferase Reporter Assays

HBx expression vector (genotype D), pCI-HBx or pCI-GST-HBx was constructed by cloning GST-HBx or HBx from a pGEX-HBx vector into pCI-neo (Promega, Tokyo, Japan). HeLa cells were seeded on 6-well plate at a density of 4×10^5^ cells/well. After 24 h, the cells were co-transfected with 0.2 mg of pCI-neo, pCI-HBx, or pCI-GST-HBx, and 0.19 mg of PathDetect Cis-Reporting Systems pAP-1-Luc, or pNF-κB-Luc (Agilent Technologies, Tokyo, Japan) as well as 0.01 mg of pGL4.74 [hRluc/TK] (Promega) for expression control using Effectene transfection reagent (Qiagen, Tokyo, Japan) according to the instructions. Luciferase assay was performed 48 h after transfection using PicaGene Dual Sea Pansy Luminescence Kit (Toyobo, Osaka, Japan) and measured by Lumistar Optima (BMG labtec, Saitama, Japan) according to the instructions. HepG2 cells were seeded on 24-well plate. After 24 h, the cells were transfected with 0.25 mg of pCI-neo or pCI-HBx, 0.25 mg of Fluc vector, and 0.01 mg of Rluc vector using FuGENE HD Transfection Reagent (Promega) according to the instructions. The compound was added 24 h after the transfection and the luciferase assay was performed 72 h after that.

### 4.5. Surface Plasmon Resonance Imaging

All the assays and analyses were performed by Plexera LLC. The binding activity was measured by surface plasmon resonance imaging (SPRi) using a PlexArray HT system (Plexera LLC). The FDA1018 drug library chip consists of an array of 1018 U.S. FDA-approved drugs were ultraviolet-linked on the surface of a chip [28,29] and used for screening of compounds that bind to the HBx protein. Phosphate-buffered saline was used for a running buffer and 10 mM glycine hydrochloride (pH 2.0) for a regeneration buffer. 25, 50, or 100 nM of GST-HBx protein were injected on the chip surface at a flow rate of 2 ml/sec in order, and the SPRi signal was measured at 25 °C. FK506-binding protein 12 (FKBP12) was used for a positive control (Supplementary Figure S1). The data were analyzed by Plexera Data Analysis Software based on Langmuir adsorption isotherm, and determination of the dissociation constant, dissociation rate constant, and association rate constant was carried out.

### 4.6. HBV Infection

HBV infection assay was performed according to the previous study [25]. On day 0, 5 genome equivalent per cell of genotype C HBV (GenBank ID: AB246345) was added on each well of a 24-well plate with 4% polyethylene glycol 8000 (Promega). At the same time, compounds were added into the culture medium. Dimethyl sulfoxide (DMSO) was used as a control. The medium and the compound were changed on day 1 and 3 and every 3 to 4 days after that.

### 4.7. HBV DNA Quantification

HepG2.2.15.7 cells were seeded on the 6-well plate at a concentration of 6 × 10^5^ cells/well. After 24 h, compounds or DMSO control were added and changed every 3 days up to 9 days. At day 9, the culture medium was collected. The HBV DNA level was measured by a company (SRL, Tokyo, Japan) by Cobas Taqman HBV test (Roche Diagnostics K.K., Tokyo, Japan) and adjusted for cell viability determined as described below. HBV DNA level was expressed as fold change compared to that in DMSO control which was set as 1. The culture medium from HBV-infected hepatocytes was measured for HBV DNA and expressed as log IU/mL.

### 4.8. HBsAg Quantificatio

HBsAg was measured by chemiluminescent enzyme immunoassay (CLEIA) (Fujirebio, Tokyo, Japan) and expressed as fold change compared to DMSO control.

### 4.9. Real-Time PCR

Cellular RNA was extracted using an RNeasy mini kit (Qiagen) and reverse-transcribed using a Primescript RT reagent kit (Takara, Shiga, Japan) according to the instructions. Real-time PCR was performed with a SYBR green PCR mastermix as previously described [30] using a StepOne real-time PCR system (Applied Biosystems, Tokyo, Japan). The data were normalized by Glyceraldehyde-3-phosphate dehydrogenase and expressed as fold difference relative to a control sample. Cellular DNA was extracted using QIAamp DNA mini kit (Qiagen). The amount of cccDNA was determined by real-time PCR using a Taqman fast advanced mastermix (ThermoFisher, Tokyo, Japan). The primers and probes were as previously described [25,31]. Human actin beta endogenous control (ThermoFisher) was used for normalization. The data were expressed as fold difference relative to a control sample.

### 4.10. Cell Viability Assay

HepG2.2.15.7 cells were seeded on the 96-well plate at a concentration of 2 × 10^3^ cells/well and treated as described above. At day 9, cell viability was determined by tetrazolium-based assays using Cell Counting Kit-8 (Dojinkagaku, Tokyo, Japan) or CellTiter 96 AQueous One Solution Cell Proliferation Assay (Promega) according to the instruction. The cell viability of the PXB cells was determined as described above.

### 4.11. Statistical Analysis

The data are expressed as the mean ± standard deviation. Statistical analysis was performed using the Student’s *t*-test. *p* < 0.05 was considered significant.

## Figures and Tables

**Table 1:** Hit compounds showing HBx binding signal by SPRi screening.

K_D_ Rank	Drugs	K_D_ (M)	Function	Cmax * (uM)	HBx Genotype
1	Tranilast	1.35 × 10^−12^	Anti-allergic	67	B/D
2	Domiphen Bromide	2.88 × 10^−11^	Antimicrobial	NA	D
3	Azithromycin	4.67 × 10^−11^	Anti-biotic	0.98	B/D
4	Alexidine hydrochloride	4.95 × 10^−11^	Antimicrobial	NA	D
5	Ammonium glycyrrhizinate	7.56 × 10^−10^	Anti-allergic	133	D
6	Valsartan	9.12 × 10^−10^	Angiotensin II receptor blocker	12.1	B/D

In total, 6 compounds with K_D_ values less than 1nM are shown. K_D_ values are based on HBx genotype D. * values are based on the prescribing information. NA, not available.

## Data Availability

Not applicable.

## Supplementary Materials

The following supporting information can be downloaded at: https://www.mdpi.com/article/10.3390/ijms231912015/s1.
