# Supplementary material for: Antiviral Compounds Screening Targeting HBx Protein of the Hepatitis B Virus
Source: Int J Mol Sci. 2022 Oct 10;23(19):12015. doi: 10.3390/ijms231912015 (PMC9569680; doi:10.3390/ijms231912015)
Supplement: Supplementary file 1 [file ijms-23-12015-s001.zip › ijms-1940271-supplementary.pdf]

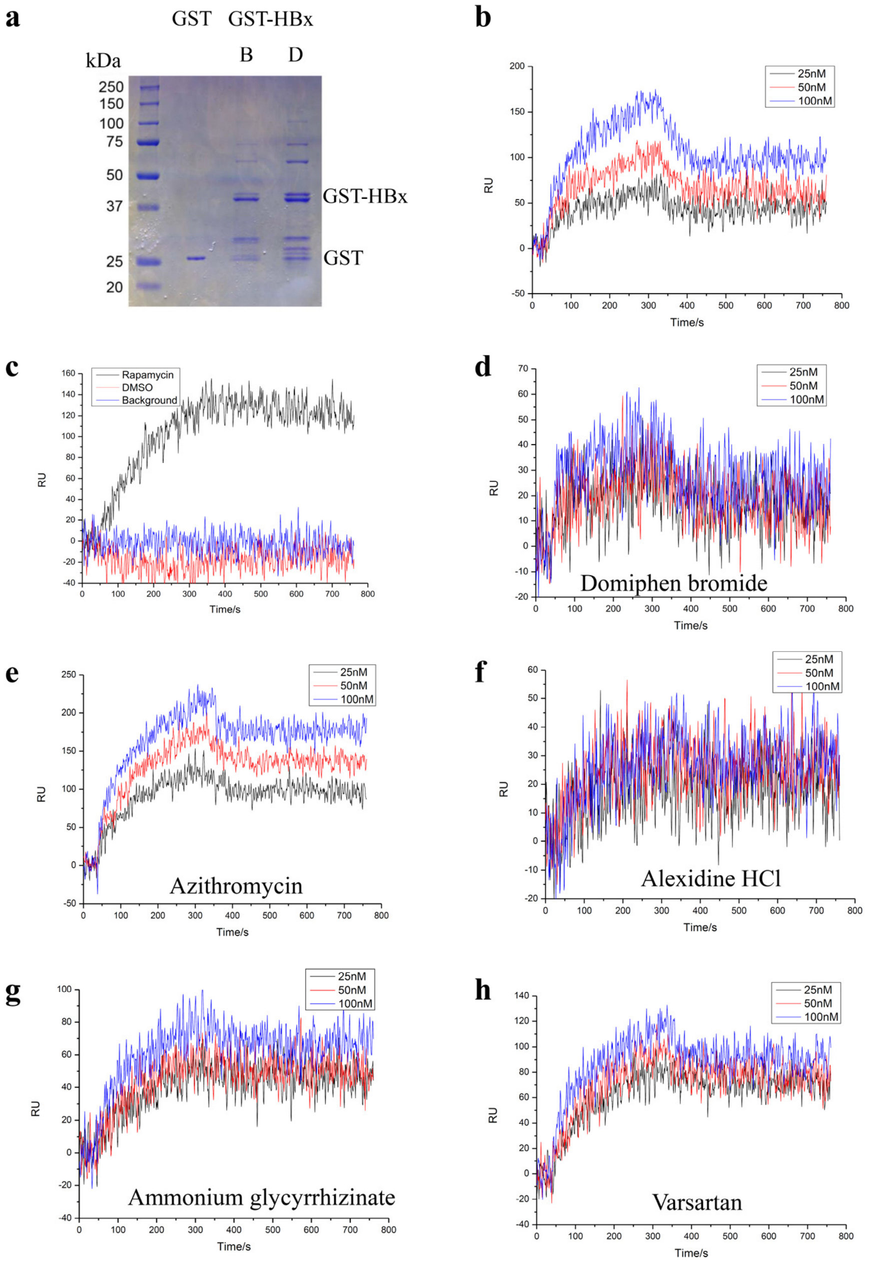

Supplementary Figure S1. Screening of GST-HBx binding compounds.

a, CBB staining of purified GST, GST-HBx (genotype B), and GST-HBx (genotype D). b, Serial dilutions of GST-HBx (genotype B) protein binding to top hit compound, tranilast. c, SPRi sensogram showing the binding of FKBP12 protein to rapamycin immobilized on the surface of a chip as a positive control. DMSO was immobilized as a negative control. d-h, SPRi sensogram showing the binding interaction of each comound to GST-HBx (genotype D).

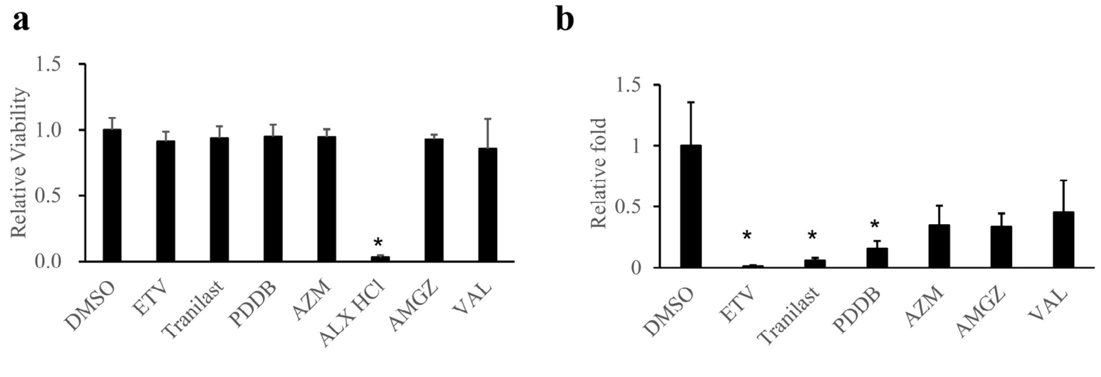

Supplementary Figure S2. Effect of compounds on anti-HBV activity in HepG2.2.15.7 cells. a, Cells were treated with compounds at the concentration of 10 μM. After 9 days, cell viability was determined by cell proliferation assay. Data were expressed as mean and standard deviation (n=8). Alexidine hydrochloride (ALX HCl) was cytotoxic therefore excluded from further evaluation. b, HBV DNA in the medium at 9 days was quantified by real-time PCR and adjusted for cell viability. The value of DMSO control was set as 1. Three compounds decreased HBV DNA significantly: entecavir (ETV, positive control), tranilast, and domiphen bromide (PDDB). Domiphen bromide was found to be cytotoxic in other liver cell line, HuH7 (data not shown), therefore excluded from further evaluation. Two independent experiments were performed in duplicate and expressed as mean and standard deviation. * $p<0.05$ compared to control.

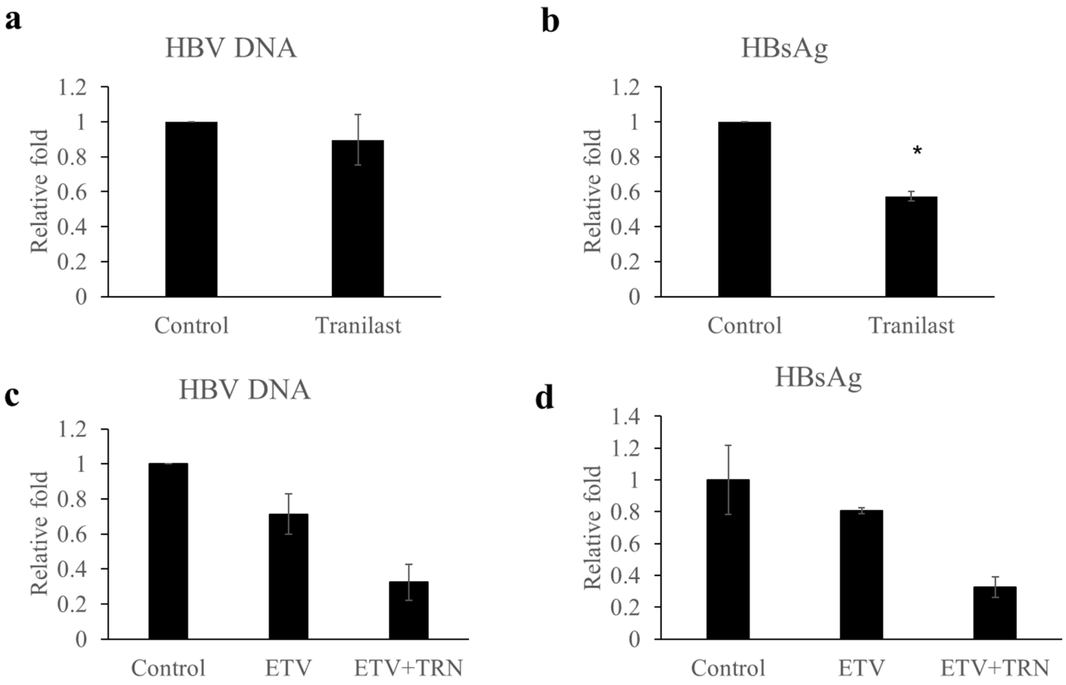

Supplementary Figure S3. Effect of tranilast on anti-HBV activity in HBV-infected hepatocytes.

a-d: Human hepatocytes, PXB cells were infected with HBV and cultured. Tranilast (TRN, 100 μM) was added on day 28, day 34 and day 38. Entecavir (ETV, 0.1μM) was added on day 34 and day 38 with or without tranilast. Note that peak plasma concentration of entecavir is 0.02-0.04 μM in the clinical setting. On day 34 (a, b) or day42 (c, d), HBV DNA in the medium, HBsAg in the medium was measured. Experiments are performed in duplicate. * $p<0.05$ compared to control.

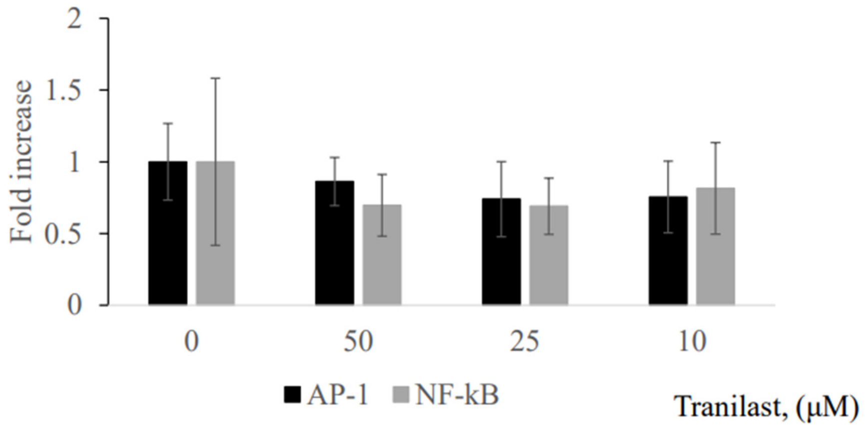

Supplementary Figure S4. Effect of tranilast on transcriptional activating function of HBx.

HepG2 cells were transfected with pCI-HBx or pCI-neo as well as indicated firefly luciferase vector and pGL4.74[hRluc/TK] control vector. Tranilast was added at the indicated concentration 24 hours after transfection. After 3 days, firefly luciferase activity was measured and corrected for the Rluc expression. Luciferase activity of cells with HBx was expressed as fold increase compared to those without HBx. Assays were performed in quadruplicate and expressed as mean and standard deviation of duplicate experiments.

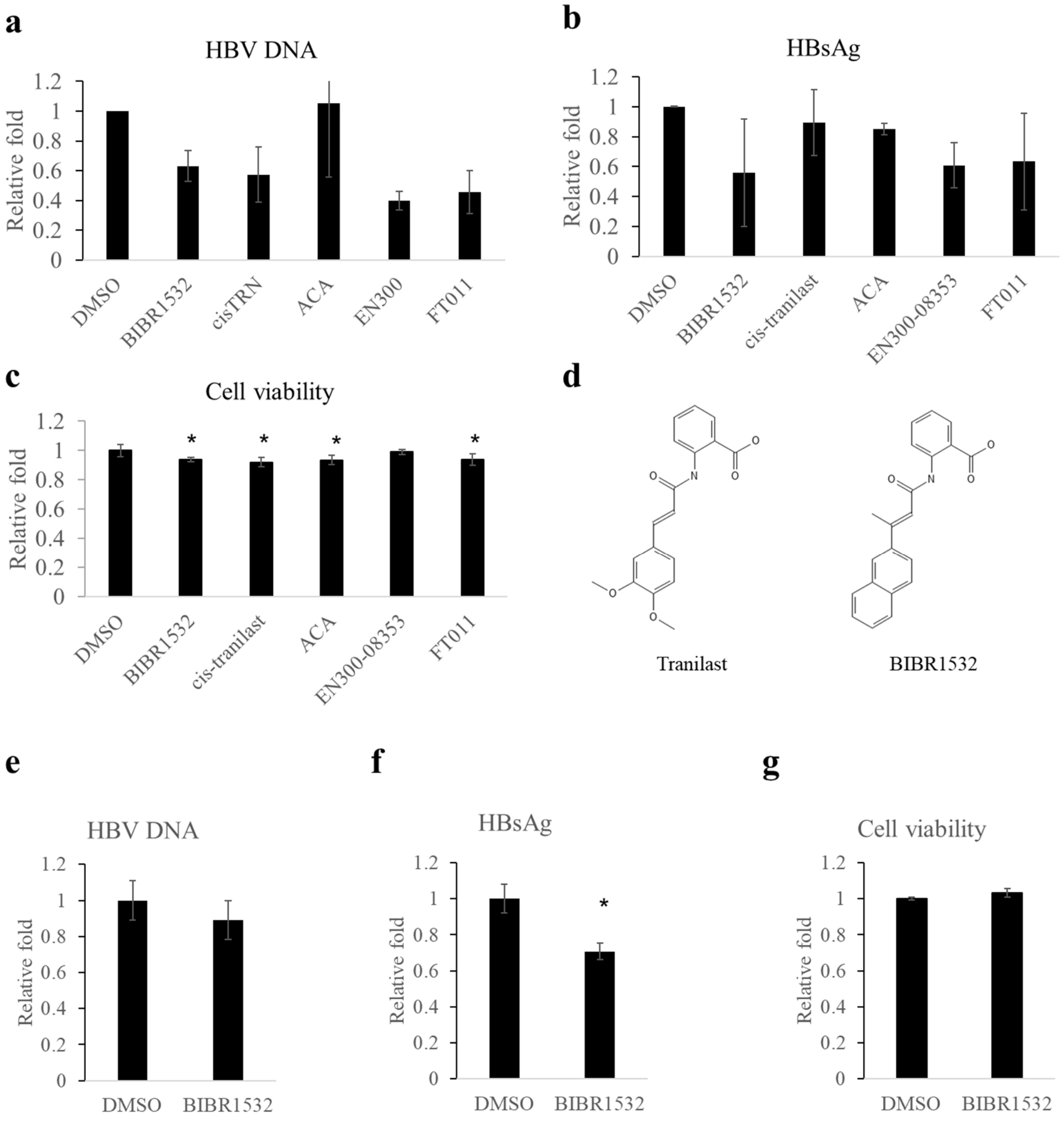

Supplementary Figure S5. Effect of tranilast analogs on HBV DNA replication.

a-c, HepG2.2.15.7 cells were treated with each compound at the concentration of 10 or 50 μM. After 9 days, HBV DNA and HBsAg level in the medium was quantified by real-time PCR (n=2). c, Cell viability was determined by cell proliferation assay (n=6). Data was combined and expressed as mean and standard deviation. * $p<0.05$ compared to control. d, Chemical structures of compounds. e-g, Human hepatocytes, PXB cells were infected with HBV on day 0 and cultured. On day 14, 10 μM of BIBR1532 was added for 7 days. On day 21, HBsAg level and HBV DNA level in the culture medium was determined. Cell viability on day 21 was

determined by cell proliferation assay. Data were expressed as mean and standard deviation (n=4). * $p<0.05$ compared to control.

Supplementary Table S1. Hit compounds against genotype B HBx by SPRi screening

	Drugs	K_D (M)	kon (M-1 s-1)	koff (s-1)
1	Tranilast	1.84×10^{-10}	5.62×10^{4}	1.03×10^{-5}
2	Nateglinide	1.11×10^{-09}	9.85×10^{3}	1.09×10^{-5}
3	Azithromycin	1.31×10^{-09}	3.38×10^{4}	4.41×10^{-5}
4	Azithromycin dihydrate	12.38×10^{-09}	1.22×10^{4}	1.69×10^{-5}
5	Ketoprofen	1.48×10^{-09}	7.14×10^{3}	1.06×10^{-5}
6	Valsartan	7.94×10^{-09}	8.96×10^{4}	7.11×10^{-4}
7	Novobiocin sodium	2.99×10^{-08}	9.64×10^{4}	2.88×10^{-3}
8	Deferasirox	2.73×10^{-6}	1.83×10^{2}	5.00×10^{-4}

Supplementary Table S2. Hit compounds against genotype D HBx by SPRi screening

	Chemical name	K_D (M)	kon (M-1 s-1)	koff (s-1)
1	Tranilast	1.35×10^{-12}	1.94×10^{5}	2.61×10^{-7}
2	Domiphen Bromide	2.88×10^{-11}	1.05×10^{5}	3.03×10^{-6}
3	Azithromycin	4.67×10^{-11}	8.92×10^{4}	4.17×10^{-6}
4	Alexidine hydrochloride	4.95×10^{-11}	1.58×10^{5}	7.83×10^{-6}
5	Ammonium glycyrrhizinate	7.56×10^{-10}	1.43×10^{5}	1.08×10^{-4}
6	Valsartan	9.12×10^{-10}	1.86×10^{5}	1.70×10^{-4}
7	Azithromycin dihydrate	1.15×10^{-09}	8.51×10^{4}	9.82×10^{-5}
8	Betaxolol	1.28×10^{-09}	7.71×10^{3}	9.85×10^{-6}
9	Tilmicosin	1.3×10^{-09}	6.77×10^{4}	8.79×10^{-5}
10	Candesartan	1.33×10^{-09}	1.58×10^{5}	2.10×10^{-4}
11	Furosemide	1.34×10^{-09}	8.11×10^{4}	1.09×10^{-4}

12	Deferasirox	1.4×10^{-09}	9.58×10^{4}	1.34×10^{-4}
13	Nateglinide	2.31×10^{-09}	6.91×10^{4}	1.60×10^{-4}
14	Azilsartan	2.38×10^{-09}	1.46×10^{5}	3.48×10^{-4}
15	Suprofen	2.75×10^{-09}	1.12×10^{5}	3.08×10^{-4}
16	Mycophenolic	4.25×10^{-09}	7.66×10^{4}	3.26×10^{-4}
17	Glipizide	4.49×10^{-09}	4.51×10^{4}	2.03×10^{-4}
18	Olmesartan medoxomil	5.37×10^{-09}	1.08×10^{5}	5.81×10^{-4}
19	Pranoprofen	5.58×10^{-09}	5.23×10^{4}	2.92×10^{-4}
20	Eltrombopag	8.46×10^{-09}	4.55×10^{4}	3.85×10^{-4}
21	Glimepiride	9.81×10^{-09}	3.73×10^{4}	3.66×10^{-4}
22	Sulfisoxazole	9.88×10^{-09}	5.15×10^{4}	5.09×10^{-4}
